# Self-Report Measurement of Well-Being in Autistic Adults: Psychometric Properties of the PERMA Profiler

**DOI:** 10.1089/aut.2022.0049

**Published:** 2023-12-12

**Authors:** Luke P. Grosvenor, Cheryl L. Errichetti, Calliope Holingue, Joan B. Beasley, Luther G. Kalb

**Affiliations:** ^1^ Department of Mental Health, Johns Hopkins Bloomberg School of Public Health, Baltimore, Maryland, USA.; ^2^ Center for Autism and Related Disorders, Kennedy Krieger Institute, Baltimore, Maryland, USA.; ^3^ Institute on Disability, University of New Hampshire College of Health and Human Services, Durham, New Hampshire, USA.

**Keywords:** positive psychology, well-being, autistic adults, mental health, autism spectrum disorder

## Abstract

**Background:**

Studies of positive psychology and emotional well-being have broadened our understanding of mental health. However, mental health research involving autistic adults has been largely deficit-focused. Few studies have examined well-being using established positive psychological frameworks.

**Methods:**

This study examined the psychometric characteristics of the PERMA Profiler, a 23-item questionnaire that measures well-being across five subscales (Positive emotion, Engagement, Relationships, Meaning, and Accomplishment), in a sample of 517 autistic adults ages 18–84 years (*M* = 39.5, standard deviation [SD] = 13.3). Reliability (internal consistency), structural validity (via confirmatory factor analysis including bifactor modeling), and concurrent validity were examined.

**Results:**

The PERMA Profiler mean (SD) well-being score was 5.4 (SD = 1.7), which is notably lower than the mean of 7.0 previously found in nonautistic samples. Subscale scores were highest for Engagement (*M* = 6.8; SD = 1.9), followed by Accomplishment (*M* = 5.6; SD = 2.2), Relationships (*M* = 5.2; SD = 2.6), Meaning (*M* = 5.2; SD = 2.7), and Positive emotion (*M* = 5.0; SD = 2.4). Factor analyses revealed strong psychometrics (Cronbach's *α* = 0.93; Comparative Fit Index = 0.94; Tucker–Lewis Index = 0.97; root mean square error of approximation = 0.08; standardized root mean residual = 0.05) and superior fit of the bifactor model, supporting a general factor for conceptualizing well-being as opposed to a five-factor model. PERMA well-being and subscale scores were significantly correlated (*p* < 0.001) with mental health conditions and life satisfaction.

**Conclusion:**

These findings support use of an adapted version of the PERMA Profiler in mental health research to evaluate well-being among autistic adults. Similar to studies with nonautistic populations, the Engagement measure may not capture the experiences of the autistic population and further refinement is needed. Follow-up research should represent a more diverse autistic population, collaborate with autistic coinvestigators, and explore potential correlates of well-being (such as social stigma) while using the PERMA Profiler.

## Community brief

**Why is this an important issue?:**

“Well-being” captures a state of comfort, health, and happiness and is more than just the absence of disease or negative feelings. Only a few studies to date have focused on the emotional well-being of autistic adults. The positive psychology-based PERMA Profiler (named after the five subscales: Positive emotions, Engagement, Relationships, Meaning, and Accomplishment) is a brief self-report measure for rating well-being using 23 questions. It has been widely used for studying well-being in nonautistic populations. However, no studies have tested how well the PERMA Profiler measures well-being among autistic adults.

**What was the purpose of this study?:**

The goal was to evaluate the PERMA Profiler using data from a group of autistic adults living in the United States. This was the first study that tested how well the PERMA Profiler measures the well-being of autistic adults, both overall and across the five subscales.

**What did the researchers do?:**

First, we collected responses to the PERMA Profiler and other questions from more than 500 autistic adults. Then, we tested whether the PERMA Profiler measures well-being consistently and if the measure performs as expected (i.e., did it measure what it is supposed to measure?). We also compared well-being scores with the mental health (anxiety and depression) and life satisfaction of the study participants.

**What were the results of the study?:**

The average overall well-being score was 5.4 out of 10, with higher values indicating experiencing well-being more often. Notably, the average well-being score was 7.0 in unrelated, previous studies of nonautistic adults). The PERMA Profiler measured well-being consistently in our sample. The overall well-being scores and each of the five subscale scores were related to mental health and life satisfaction as expected. The “Engagement” subscale did not perform as well as the others in our sample, which was consistent with the findings of studies with nonautistic adults.

**What do these findings add to what was already known?:**

This is the first study to evaluate the effectiveness of the PERMA Profile or any measure of self-reported well-being in a sample of autistic adults. These results can help future researchers determine how best to study well-being, specifically in autistic adults. These findings point to important changes that might be made to the PERMA Profiler before it is used in future research.

**What are potential weaknesses in the study?:**

Autistic adults were not involved in the development of the PERMA Profiler. Therefore, the measure may need to be changed by teams involving autistic coinvestigators before use with other samples. Second, our findings may not represent larger, more diverse groups of autistic adults because most participants were White (85%), well-educated (more than 80% completed at least some college), and did not have intellectual disability. Lastly, the study was conducted during the COVID-19 pandemic, which likely impacted the well-being of participants.

**How will these findings help autistic adults now or in the future?:**

Our study provides preliminary support for the PERMA Profiler and suggests next steps for improving it and other measures of well-being before expanding use with autistic adults. Our findings may help the field of autism research develop new measures for understanding and improving well-being. The strengths and weaknesses of the PERMA Profiler that we identified can inform future strengths-based research involving autistic adults.

## Introduction

Autistic adults experience significantly higher rates of mental^1^ and physical health conditions^2–4^ compared with nonautistic adults. Furthermore, lack of treatment for these conditions leads to a premature mortality rate among autistic persons.^5,6^ Autistic adults also experience limited access to services, recreational activities, and employment and education opportunities.^7,8^ These factors, combined with the growing population of autistic adults living in the United States,^9^ underscore a need to improve the overall health of this population. However, despite the World Health Organization's long-standing emphasis that health is, “a state of complete physical, mental and social well-being and not merely the absence of disease or infirmity,”^10^ emotional well-being in autistic adults has not been a focus of health research.

Treatment approaches in all forms of health care have increasingly emphasized positive practices to improve physical and mental health. A growing body of multidisciplinary research in nonautistic populations has shown that subjective well-being is independently associated with improved social and occupational wellness and lower rates of substance use, cardiovascular disease, and mortality rate.^11–13^ A singular focus on psychopathology or the treatment of mental illness falls short of promoting mental health and attending to the strengths in autistic adults. A preponderance of the “well-being” research on caregiver experiences with autistic children has in fact focused on the stress and psychopathology associated with caregiving.^1,14,15^ For autistic adults, there have only been a few studies of self-reported well-being (reviewed below, Introduction), while most others have relied on caregiver- or proxy-report measures. This is likely due, in part, to the lack of validated and accessible services and research tools, including self-report measures of well-being, in autistic persons.^16,17^

Three recent studies of self-reported well-being in autistic adults found associations between higher overall well-being and greater connectedness to social groups,^18^ lower levels of aloofness/social anhedonia,^19^ and lower ratings of depressive symptoms.^20^ In a study of 184 autistic adults, those who reported feeling greater social connectedness with their families and to a higher number of distinct social groups (such as hobby groups, work and other peer groups) had not only lower depressive symptom ratings, but significantly higher overall well-being scores.^18^ Another recent study involving 227 autistic adults reported that lower levels of social aloofness, as measured by the Broad Autism Phenotype Questionnaire (BAPQ), were correlated with higher overall well-being and that this association was partially mediated by perceived level of crisis support (e.g., the number of people they could turn to in personal crisis).^19^

A third, longitudinal study of 36 currently employed autistic adults found that higher overall mental well-being scores were protective against the development of future depression.^20^ These studies measured subjective well-being using the 7- or14-item version of the Warwick–Edinburgh Mental Well-Being Scale,^21,22^ which was validated in a sample of predominantly nonautistic/neurotypical adults selected from universities in the United Kingdom.^23^ These and other researchers have highlighted a need for validated measures that can be used in large, diverse samples of autistic adults for epidemiologic (e.g., identifying prevalence and correlates) and intervention (e.g., outcome measures) purposes.^20^ While we have reviewed only studies of self-report well-being measures here, there have been considerable efforts that included autistic adults to develop positive psychological measures of other domains, such as quality of life.^24^

Numerous definitions of subjective well-being exist in the field of positive psychology, often with focuses on the affective (e.g., positive emotion) and evaluative (e.g., meaning, purpose) components.^25^ Martin Seligman, a founder and pioneer of positive psychology, defined well-being in terms of five pillars: Positive emotion, Engagement, Relationships, Meaning, and Accomplishment, or “PERMA.”^26^ From this model, Bulter and Kern^27^ developed the PERMA Profiler, a 23-item self-report measure of well-being that quantifies each of the five pillars, providing subscale scores in addition to a score for overall well-being.

Items used in the PERMA Profiler were first selected from testing within a sample of more than 7000 adults and the measure subsequently demonstrated high reliability and validity using data from 8 samples totaling more than 30,000 adults recruited from 12 regions around the world.^27^ While psychometrically sound in nonautistic/neurotypical adults, it is unknown whether the PERMA Profiler is a valid and reliable measure for use with autistic adults.

The objective of this study was to expand well-being research among autistic adults by assessing the psychometric properties (reliability, structural validity, and convergent and divergent validity) of the PERMA Profiler. We hypothesize that the measure will be psychometrically valid, thus providing avenues to promote strengths-based research into the correlates and predictors of well-being in autistic adults.

## Materials and Methods

### Participants and procedures

We recruited the sample of autistic adults to join the study through Simons Powering Autism Research for Knowledge (SPARK; sparkforautism.org^28^), an online research match program. After enrolling and agreeing to share their data, participants completed a brief online questionnaire about their general health and well-being. All participants were entered into a raffle to win a gift card as compensation and the study was approved by the Johns Hopkins School of Medicine Institutional Review Board.

We collected the cross-sectional data online over a period of 3 weeks beginning in August 2021. The SPARK research match program sent email invitations in two waves to 3044 autistic individuals enrolled in SPARK. Eligible study invitees were adults ages 18–100 years who self-identified as autistic and lived anywhere within the United States. SPARK sent automated reminder emails 3 and 5 days after the initial invitation to participate. The 68-item custom survey included the 23-item PERMA Profiler (detailed below, Measurements) and questions about sociodemographic, mental health, education, employment, and other characteristics. Participants took ∼10–15 minutes to complete the survey.

### Measurements

#### Sociodemographic characteristics

In the first part of the survey, participants provided information about their age, living situation (e.g., alone/independent, with parent(s)/caregiver(s), shared home), highest education completed, employment status, marital status, assigned sex at birth, gender identity, race and ethnicity.

#### Well-being

For each of the 23 items of the PERMA Profiler, participants reported how often or to what extent they felt a particular way (e.g., *How often do you feel joyful?*). Likert-scale responses ranged from 0 (Never or Not at all) to 10 (Always or Completely). Three items corresponded to each of the five PERMA subscales: Positive emotion (experience of positive emotions), Engagement (being immersed in life pursuits), Relationships (having satisfying relationships with others), Meaning (working toward a bigger goal), and Accomplishment (regularly achieving successes). Six additional items within the PERMA Profiler were used to measure negative emotions (NE; three items) and overall physical health (PH; three items). The PERMA Profiler is freely available online for use (see the Data and Questionnaire Availability section for a link to the full questionnaire and scoring guidelines).

The final item of the custom survey invited participants to share open-text responses related to their well-being. This question specifically asked, *Is there anything else you would like to tell us that we have not already asked, related to your well-being, your child/dependent's well-being (if applicable), or your family's well-being during COVID-19?*

#### Mental health

Participants reported currently experiencing anxiety and/or depression by selecting from a list of psychiatric conditions. Specifically, the question asked, *Are any of the following a problem for you? (Select all that apply).* Participants were able to endorse, *Anxiety (*e.g., *feelings of fear, worry, or panic)* and/or *Depression (*e.g., *feeling sad or not interested in things you usually enjoy)*.

#### Life satisfaction

Participants provided information on overall life satisfaction by rating their agreement with two single-item prompts. This included: (1) *The conditions of my life are excellent* and (2) *I am satisfied with my life*. Response options ranged from 0 (Strongly Disagree) to 6 (Strongly Agree).

### Analytic approach

#### Reliability

We calculated three measures of internal consistency to determine the reliability of the items included in the PERMA Profiler: Cronbach's alpha,^29^ minimum split-half reliability, and maximum split-half reliability. We generated each of these for the five PERMA subscales and the measure of overall well-being. Alpha and split-half values greater than 0.70, 0.80, and 0.90 indicated acceptable, good, and excellent reliability, respectively.

#### Structural validity

Before performing a confirmatory factor analysis (CFA) to assess the structural validity of the measure, we used Bartlett's test of sphericity^30^ and the Kaiser–Meyer–Olkin (KMO) measure of sampling adequacy^31^ to determine whether the use of CFA was appropriate for these data. Then, we performed the CFA to test the fit of the five-factor PERMA model specified by Bulter and Kern^27^ as well as a bifactor model, which tests whether PERMA can be represented as a single overall factor, rather than with five discrete factors.^32,33^ We evaluated the fit of each model using the root mean square error of approximation (RMSEA) and standardized root mean residual (SRMR). An RMSEA of 0.08 or below (and a lower 90% confidence interval bound <0.05) and an SRMR of 0.08 or lower indicate acceptable model fit, while values less than 0.06 indicate excellent fit.^34,35^

We used the Comparative Fit Index (CFI) and Tucker–Lewis Index (TLI) as additional incremental fit metrics for both models. CFI and TLI values above 0.90 are considered indicators of good model fit and above 0.95 excellent fit.^34^

Reliability of the bifactor model was assessed using Omega (scores range from 0.0 to 1.0), which represents how well the overall PERMA scores (produced from all 16 items) measure the underlying well-being construct.^36^ OmegaH and OmegaHS measure the proportion of variability in well-being explained by the general factor alone and by each of the five subscales after accounting for the general factor, respectively. OmegaH and OmegaHS values that differ greatly from Omega indicate low reliability for each of the general and specific factors. Beyond Omega, we used H-Index and factor determinacy (FD) values to evaluate model fit, with values greater than 0.80^37^ and 0.90,^38^ indicating strong performance of each factor in defining the underlying construct, respectively. Lastly, the proportion of variance explained by the general factor versus by each individual factor alone was evaluated using explained common variance (ECV) and percent uncontaminated correlations (PUC).

Values for PUC greater than 0.80 *or* less than 0.80 combined with ECV greater than 0.60 and OmegaH greater than 0.70 support a unidimensional model of well-being represented by the general factor.^39^ We accounted for missing data in the CFAs using full information maximum likelihood, where the likelihood case by case was computed using all the available measured data from that case.^40^

#### Convergent and divergent validity

We tested for the convergent and divergent validities of the PERMA Profiler using correlations between overall well-being scores, PERMA subscale scores, and measures of satisfaction with life, feelings that life is excellent, depression, and anxiety. Tetrachoric correlations were used to assess relationships between categorical variables (e.g., anxiety, depression) that are assumed to represent underlying continuous distributions.^41^ The individual PERMA items measuring loneliness and sadness were reverse-coded (e.g., a positive association demonstrates a greater PERMA score is related to higher life satisfaction and lower depression and anxiety) to facilitate interpretation. We reported psychometric properties for the PERMA Profiler in accordance with the COnsensus-based Standards for the selection of health status Measurement INstruments (COSMIN) guidelines.^42^ We performed all analyses using the *psych*,^43^ *REdaS*,^44^ *lavaan*,^45^ and *BifactorIndicesCalculator*^46^ packages in R version 4.0.3.^47^

## Results

### Participants

Participant ages ranged from 18 to 84 years (*M* = 39.5; standard deviation [SD] = 13.3). Five hundred thirty-four out of 3004 (17.5%) invited participants accepted the study invitation and 517 of the 534 (96.8%) completed the questionnaire (an invitation acceptance rate of ∼20% was consistent with other SPARK research match studies involving online questionnaires). The greatest proportion of study participants identified as female/woman (49.1%), White (84.6%), and having completed at least some college, a bachelor's degree, master's degree, or higher (82.0%). More than half of the participants (51.9%) reported being currently employed. A full summary of the sociodemographic characteristics of the study sample is presented in Table 1.

**Table 1.** Sociodemographic Characteristics of the Study Sample (*N* = 517)

Gender, *n* (%)	
Cis-female/woman	254 (49.1)
Cis-male/man	188 (36.4)
Gender nonconforming	42 (8.1)
Identify as other	14 (2.7)
Prefer not to report	11 (2.1)
Transgender male	8 (1.5)
Age group, *n* (%)	
18–24	62 (12.2)
25–34	164 (32.2)
35–44	128 (25.1)
45–54	77 (15.1)
55–64	55 (10.8)
65+	24 (4.6)
Race, *n* (%)	
White	433 (84.6)
American Indian or Alaska Native	24 (4.7)
Black or African American	21 (4.1)
Other	18 (3.5)
Asian	15 (2.9)
Native Hawaiian or other Pacific Islander	1 (0.2)
Ethnicity, *n* (%)	
Not Hispanic or Latino	460 (89.0)
Hispanic or Latino	46 (8.9)
Prefer not to report	11 (2.1)
Education level, *n* (%)	
Less high school diploma	9 (1.7)
High school diploma or equivalent (e.g., GED)	59 (11.4)
Trade (technical, vocational, or military training)	25 (4.9)
Some college	113 (21.9)
Associate's degree	47 (9.1)
Bachelor's degree	152 (29.4)
Master's degree	75 (14.5)
Professional degree	8 (1.5)
Doctoral degree	29 (5.6)
Employment	
Currently employed, *n* (%)	268 (51.9)

### Mental health and life satisfaction

A total of 359 (69.4%) participants endorsed having problems with depression and 423 (81.8%) with anxiety, which are consistent with higher rates of these disorders compared with samples of nonautistic adults.^48^

In response to the question about life satisfaction, 22.4% strongly agreed or agreed, 22.0% slightly agreed, 11.8% neither agreed nor disagreed, 13.7% slightly disagreed, and 30.2% either disagreed or strongly disagreed that they were satisfied with life. Regarding whether the conditions of their life were excellent, 24.7% strongly agreed or agreed, 22.3% slightly agreed, 12.7% neither agreed nor disagreed, 13.9% slightly disagreed, and 26.2% either disagreed or strongly disagreed.

### PERMA Profiler

**FIG. 1.**
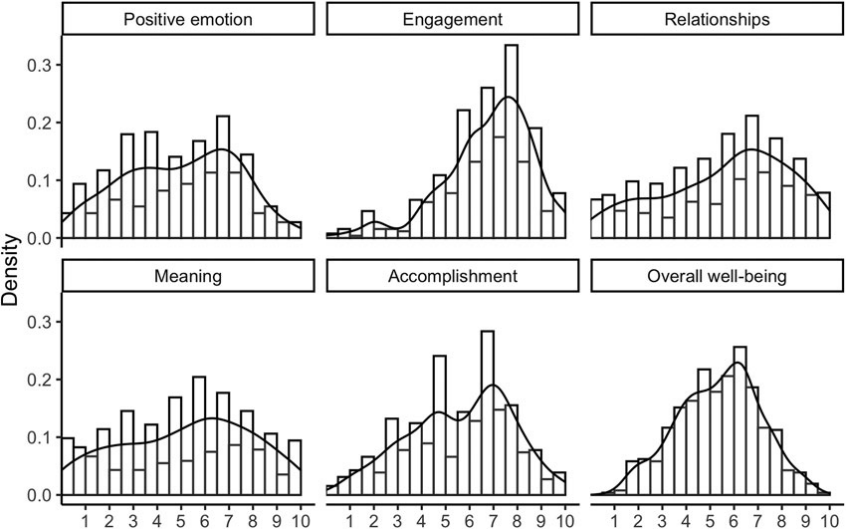
Distributions of PERMA overall well-being and subscale scores for all study participants.

The PERMA Profiler mean (SD) overall well-being score was 5.4 (SD = 1.7) and scores were highest for Engagement (*M* = 6.8; SD = 1.9), followed by Accomplishment (*M* = 5.6; SD = 2.2), Relationships (*M* = 5.2; SD = 2.6), Meaning (*M* = 5.2; SD = 2.7), and Positive emotion (*M* = 5.0; SD = 2.4). Distributions for overall well-being and each of the subscales are displayed in Figure 1. Missingness for the individual PERMA items ranged from 4% for the first Engagement item (question 2 out of 23, *How often do you become absorbed in what you are doing?*) to 9% for the third Physical Health question (question 19 out of 23, *Compared with others of your same age and sex, how is your health?*).

Of the 23 items, the proportion of missingness was significantly associated with sociodemographic variables for 3 items (E3: *How often do you lose track of time while doing something you enjoy?* H3: *Compared with others of your same age and sex, how is your health?* R3: *How satisfied are you with your personal relationships?*). A summary of item missingness and results from association tests with sociodemographic variables is included in Supplementary Table S1 and Supplementary Figure S1.

### Internal consistency

Cronbach's alpha indicated good internal consistency for four out of the five subscales of the PERMA Profiler: Positive emotion (*α* = 0.89), Relationships (*α* = 0.85), Meaning (*α* = 0.82), and Accomplishment (*α* = 0.81) and excellent internal consistency for the overall measure of well-being (*α* = 0.93). The Engagement subscale demonstrated weaker internal consistency (*α* = 0.61). The split-half reliabilities supported similar results as Cronbach's alpha, with minimum and maximum reliabilities for Engagement being the lowest (0.46 and 0.63, respectively) and high minimum and maximum split-half reliability (0.81 and 0.97, respectively) for overall PERMA well-being.

### Structural validity

Results from Bartlett's test of sphericity [*χ*^2^(253) = 4414, *p* < 0.001] and the KMO measure of sampling adequacy (KMO = 0.93) indicated adequacy of the correlation matrix to conduct CFAs. Each of the five-factor modes [*χ*^2^ (80) = 350, *p* < 0.001] and the bifactor models [*χ*^2^(89) = 311, *p* < 0.001] adequately fit the data, and model fit indices supported acceptable to excellent structural validity for the measure. Inclusion of a general or overall well-being factor in the bifactor model slightly improved model fit compared with the five-factor model [five-factor: RMSEA = 0.08 (0.072–0.090); TLI = 0.94; CFI = 0.93; SRMR = 0.05; bifactor: RMSEA = 0.07 (0.061–0.078); TLI = 0.95; CFI = 0.96; SRMR = 0.04].

Loading values onto the overall well-being factor were lowest for the Engagement subscale, with two of the three item loadings below 0.40 (E1 = 0.28; E2 = 0.79; E3 = 0.22) along with one of the Accomplishment items (A3 = 0.34). All other items loaded adequately onto the overall well-being factor (Table 2). Stratified CFAs revealed that there were no differences in model fit statistics by age group (less than vs. greater than or equal to 37 years) or gender (woman/female vs. man/male; Supplementary Table S2).

**Table 2.** Factor Loading Values from the Bifactor Model of the PERMA Profiler

Item stem	Overall	P	E	R	M	A
P1. Joy	0.81					
P2. Positive	0.84	0.46				
P3. Content	0.87					
E1. Absorb			0.73			
E2. Excite	0.79					
E3. Time			0.58			
R1. Support	0.59			0.41		
R2. Loved	0.63			0.76		
R3. Relation	0.66			0.40		
M1. Purpose	0.79				0.42	
M2. Value	0.74				0.50	
M3. Direction	0.73					
A1. Goals1	0.71					0.47
A2. Goals2	0.60					0.66
A3. Responsibilities						0.45
Overall happiness	0.91					

Item loadings below 0.40 are not shown.

P, Positive emotion; E, Engagement; R, Relationships; M, Meaning; A, Accomplishment; Number denotes item number (e.g., E2: Engagement factor, item 2).

Regarding results from the bifactor reliability and dimensionality analyses, Omega was 0.96 and OmegaH was 0.89 for the general/overall well-being factor. These were the highest among all factors and indicate that 92% (0.89/0.96) of the variance in the total PERMA scores can be explained by the general/overall factor. Differences between Omega and OmegaHS values for the five subscales were substantially different, indicating that the subscales do not contribute to variance in well-being independent of the general/overall well-being factor. For example, Omega values for Positive emotions and Relationships were 0.91 and 0.87, respectively, and OmegaHS values for each were 0.04 (4% of Omega) and 0.35 (41% of Omega). The H-Index for the overall factor was 0.96 and FD was 0.97, which, together with the comparisons of Omega with OmegaH/HS values for each factor, support a unidimensional factor for representing well-being in autistic adults.

The bifactor model is also supported by the fact that almost all items either solely loaded on the overall factor, or cross loaded (at >0.40; see Table 2). A full summary of the bifactor reliability and dimensionality statistics is included in Table 3.

**Table 3.** Bifactor Reliability and Dimensionality Statistics

Factor	ECV	Omega	OmegaH or HS	H-Index	FD
Overall	0.69	0.96	0.89	0.96	0.97
P	0.02	0.91	0.04	0.23	0.79
E	0.08	0.73	0.41	0.63	0.81
R	0.08	0.87	0.35	0.64	0.96
M	0.05	0.91	0.23	0.42	0.81
A	0.08	0.82	0.39	0.57	0.86

ECV, explained common variance; FD, factor determinacy; P, Positive emotion; E, Engagement; R, Relationships; M, Meaning; A, Accomplishment.

### Convergent and divergent validity

The overall PERMA well-being scores were positively correlated with life satisfaction (*r* = 0.76, *p* < 0.001) and agreement that life is excellent(*r* = 0.72, *p* < 0.001), and negatively correlated with anxiety (*r* = −0.20, *p* < 0.001) and depression (*r* = −0.42, *p* < 0.001), supporting convergent and divergent validity, respectively, of the PERMA profiler. Subscale scores, including those for Negative emotion and Physical health, correlated similarly with these measures and further supported the validity of the PERMA Profiler. Details supporting convergent and divergent validity are presented in Supplementary Table S3.

## Discussion

Few self-report measures exist for autistic adults and no self-report measures of well-being, to our knowledge, have been validated for this population. This study addresses that gap by demonstrating strong psychometric properties for the PERMA Profiler among a large sample of autistic adults. Taken in the context of study limitations, these findings support future use of the PERMA Profiler to gather information directly from autistic adults to study the determinants of well-being.

Factor analyses revealed that subjective well-being among autistic adults is best conceptualized by a unidimensional factor of overall well-being, instead of the five-factor PERMA model proposed by Bulter and Kern.^27^ This means PERMA well-being, in autistic adults, may be best conceptualized as an overall construct rather than as individual, constituent parts. Beyond the factor structure, the reliability, structural validity, and convergent and divergent validity of the PERMA Profiler were all high. This further supports the use of the PERMA Profiler in autistic adults. Importantly, these findings must be viewed in the context of the specific characteristics of our study sample (e.g., high level of education).

Regarding the five subscales of PERMA, the construct that demonstrated the lowest psychometrics (in particular, loadings onto each of the general well-being and subscale factors) was Engagement. This finding is consistent with the original development and validation of the PERMA Profiler^27^ and studies that have adapted it for use outside the United States.^49,50^ Poorer psychometric statistics may be produced from problems with item wording that led to inconsistencies in interpretations among our sample. Bulter and Kern^27^ chose the Engagement items from a bank of 23 that each represented one of four subdomains: Absorption, Effort, Interest, and Involvement. The three items used in the final measure specifically measure Absorption (E1 and E3) and Interest (E2).

It is possible that autistic traits, such as the presence of restricted interests and/or repetitive behaviors, contributed to poorer psychometrics for these specific Engagement items. For example, the item *How often do you become absorbed in what you are doing?* may perform differently for some autistic adults with restricted interests compared with those without, and perhaps more so compared with nonautistic adults. Autistic adult experiences of stigma and social isolation would also likely impact the extent to which they engage and become absorbed in activities, compared with the nonautistic population. In its current form, the Engagement subscale is a weaker measure than the others within the PERMA Profiler and our analyses suggest altering the items chosen to measure Engagement in autistic adults.

It will be critical to involve autistic adults in this revision process, especially considering they were not well-represented in the original PERMA validation study, and to incorporate cognitive interviewing (e.g., “think-aloud” interviewing, verbal probing)^51^ and other best practices for rewording and/or selecting new items.

Further validation of the PERMA Profiler in a large and diverse sample of autistic adults can inform approaches for measuring well-being in this population in multiple ways. First, given that the PERMA Profiler is brief and readily available (e.g., takes less than 10 minutes to complete, free for use online), our findings could encourage researchers and mental health clinicians to use first-person as opposed to proxy- or informant-reports for studying well-being whenever possible. Proxy-reports of well-being may also not be an accurate representation of the autistic experience as concordance rates have been low between proxy- and self-reported measures of quality of life^52^ and daily living skills.^53^ An over-reliance by researchers on proxy-reports also silences the voices of autistic adults by failing to learn and appreciate their perspectives and capabilities.

In addition, the PERMA Profiler should be considered for inclusion as an outcome measure in future mental health research involving autistic adults. For example, a recent trial piloted the use of a mindfulness-based stress reduction (MBSR) intervention in autistic adults and demonstrated improvements in both disability-related and autism-specific quality of life, measured using the WHO QoL Brief Version.^54^ Use of measures such as the PERMA Profiler would enable researchers to evaluate the impact of MBSR on components of well-being, such as positive emotion, that are different from general quality of life and have meaning independent of psychopathologic outcomes. Beyond MBSR, multiple clinical trials of interventions including psychological, pharmacological, mindfulness, physical activity, occupational therapy, and other treatments have found significant and sustained improvements in overall well-being and quality-of-life outcomes, including mental and physical health subscales, for treatment windows ranging from 8 weeks to 12 months.^55–58^

However, no studies to date have tested responsiveness to change over time in well-being measured using the PERMA Profiler. Given the findings from the bifactor model, any clinical trial may be best to consider changes in overall PERMA, rather than specific indices.

The findings from this study may also provide a foundation for the use of PERMA-informed positive psychological interventions among autistic adults. The average scores for overall well-being and most PERMA subscales were near the midpoint (5 out of 10) and were lowest for Positive emotion and Meaning. The overall well-being score for our sample (5.4) was considerably lower than in previous studies that validated the questionnaire in nonautistic samples (7.0).^27^ However, definitive interpretations of any differences should be made with caution because our study did not include a nonautistic comparison sample and because the construct of “well-being” may differ considerably between populations.

It is also important to note that lower scores may not be attributable to the autistic experience, but rather to the conditions under which people who are autistic people live within society. Given the societal stigma associated with autism, it is not surprising that well-being scores are lower. Inclusive research that represents the voices of the autistic community is needed.

There are notable limitations of the present study. First, all participants self-reported their diagnoses of autism, which may raise concerns about diagnostic validity. However, online, caregiver-reported diagnoses of autism have demonstrated high validity when compared with the gold-standard clinical diagnoses,^59^ and parent/caregiver- and self-reports of diagnoses for autistic adults have shown high concordance.^53^ In addition, by relying on self-identification instead of requiring proof of diagnosis, we may have been able to represent a wider range of autistic identities.

Second, a significant majority (over 80%) of our sample was well-educated, White, and non-Hispanic, and therefore not representative of the greater autistic adult population. This is due mostly to our recruitment method as the demographic characteristics of our sample are similar to those of the larger SPARK cohort. While our sample size was sufficient to support psychometric validation of the measure,^60^ future research would benefit from recruitment of and engagement with a more sociodemographically and otherwise diverse sample. For example, future well-being research should include more individuals with intellectual disability (we did not collect information related to intellectual disability from our sample). Recruiting and engaging people who use or have used services relating to intellectual and other disabilities would inform revisions to improve both the accuracy and accessibility of the PERMA Profiler.

Another limitation is that we did not formally evaluate the content validity of the PERMA Profiler by defining “well-being” in collaboration with autistic adults before measurement. Lack of content validity is an inherent drawback of the “top-down” approach of using a previously validated measure in a different study population. We recognize that taking a “bottom-up” approach will be necessary to define the most accurate well-being constructs for autistic adults. There may be meaningful differences between autistic and nonautistic well-being or flourishing that we were unable to capture, similar to aspects of autistic quality of life differing greatly from nonautistic quality of life.^61^

Finally, we collected our data during the COVID-19 pandemic, which likely had wide-ranging impacts on the well-being of the study participants. Several participants shared details about changes to their overall, physical and mental health in responses to the open-text question about COVID impacts on their well-being at the end of the survey. While several described the negative impacts, multiple participants highlighted emotional and mental health benefits from COVID-related changes to their lifestyle (e.g., reduced feelings of stress and anxiety due to experiencing limited and more intentional social interactions). Formal qualitative analyses of these responses may be conducted in future work but beyond the scope of this article.

Self-reported measurement of well-being of autistic adults using the PERMA Profiler is a step toward expanding self-report measurement and informing positive psychological interventions for this population. Future work should test and adapt the PERMA Profiler and other measures of well-being in more diverse samples. This work should advance humanistic methods that promote the enrichment of positive emotion, engagement, relationships, meaning, and accomplishment while collaborating with and incorporating feedback from autistic adults and persons with disabilities.

## Supplementary Material

Supplemental data

Supplemental data

Supplemental data

Supplemental data

## Data and Questionnaire Availability

Please contact the corresponding author if you are interested in working with the data reported in this article. Data will be made available upon reasonable request through SFARI Base at https://base.sfari.org/ The PERMA Profiler is freely available for use, with the full questionnaire and scoring guidelines here: https://www.peggykern.org/uploads/5/6/6/7/56678211/the_perma-profiler_101416.pdf
