# Supplementary material for: Self-Report Measurement of Well-Being in Autistic Adults: Psychometric Properties of the PERMA Profiler
Source: Autism Adulthood. 2023 Dec 12;5(4):401–10. doi: 10.1089/aut.2022.0049 (PMC10726181; doi:10.1089/aut.2022.0049)
Supplement: Supplemental data [file Suppl_TableS1.docx]

**Table S1**. Item-level missingness for all 23 items of the PERMA Profiler and p-values from chi-square tests of differences across levels of observed sociodemographic variables

| Item | N  missing | %  missing | Age  Group | Sex | Gender | Race | Ethnicity | Education | Employed |
| --- | --- | --- | --- | --- | --- | --- | --- | --- | --- |
| q45_informant_wb_goals | 39 | 7.54 | ns | ns | ns | ns | ns | ns | ns |
| q46_informant_wb_absorb | 20 | 3.87 | ns | ns | ns | ns | ns | ns | ns |
| q47_informant_wb_joy | 42 | 8.12 | ns | ns | ns | ns | ns | ns | ns |
| q48_informant_wb_anxious | 32 | 6.19 | ns | ns | ns | ns | ns | ns | ns |
| q49_informant_wb_accomplish_goals | 48 | 9.28 | ns | ns | ns | ns | ns | ns | ns |
| q50_informant_wb_overall_health | 49 | 9.48 | ns | ns | ns | ns | ns | ns | ns |
| q51_informant_wb_purpose | 42 | 8.12 | ns | ns | ns | ns | ns | ns | ns |
| q52_informant_wb_support | 28 | 5.42 | ns | ns | ns | ns | ns | ns | ns |
| q53_informant_wb_value | 37 | 7.16 | ns | ns | ns | ns | ns | ns | ns |
| q54_informant_wb_excite | 37 | 7.16 | ns | ns | ns | ns | ns | ns | ns |
| q55_informant_wb_lonely | 32 | 6.19 | ns | ns | ns | ns | ns | ns | ns |
| q56_informant_wb_satis_ph | 36 | 6.96 | ns | ns | ns | ns | ns | ns | ns |
| q57_informant_wb_positive | 48 | 9.28 | ns | ns | ns | ns | ns | ns | ns |
| q58_informant_wb_angry | 44 | 8.51 | ns | ns | ns | ns | ns | ns | ns |
| q59_informant_wb_responsibilities | 23 | 4.45 | ns | ns | ns | ns | ns | ns | ns |
| q60_informant_wb_sad | 38 | 7.35 | ns | ns | ns | ns | ns | ns | ns |
| q61_informant_wb_lose_time | 28 | 5.42 | ns | ns | ns | ** | ns | ns | ns |
| q62_informant_wb_health_compare | 49 | 9.48 | ns | * | ns | ns | ns | * | ns |
| q63_informant_wb_loved | 23 | 4.45 | ns | ns | ns | ns | ns | ns | ns |
| q64_informant_wb_direction_life | 32 | 6.19 | ns | ns | ns | ns | ns | ns | ns |
| q65_informant_wb_satis_relation | 36 | 6.96 | ns | ns | ns | * | ns | ns | ns |
| q66_informant_wb_content | 36 | 6.96 | ns | ns | ns | ns | ns | ns | ns |
| q67_informant_wb_happy_overall | 43 | 8.32 | ns | ns | ns | ns | ns | ns | ns |

ns= not statistically significant.
