# Supplementary material for: Self-Report Measurement of Well-Being in Autistic Adults: Psychometric Properties of the PERMA Profiler
Source: Autism Adulthood. 2023 Dec 12;5(4):401–10. doi: 10.1089/aut.2022.0049 (PMC10726181; doi:10.1089/aut.2022.0049)

**Figure S1.** Heat map displaying differences in proportions of missingness for all 23 PERMA items, in this example stratified by biological sex. Differences between groups were significant for items 60, "q60_informant_wb_sad" and 62, “q62_informant_wb_health_compare.”


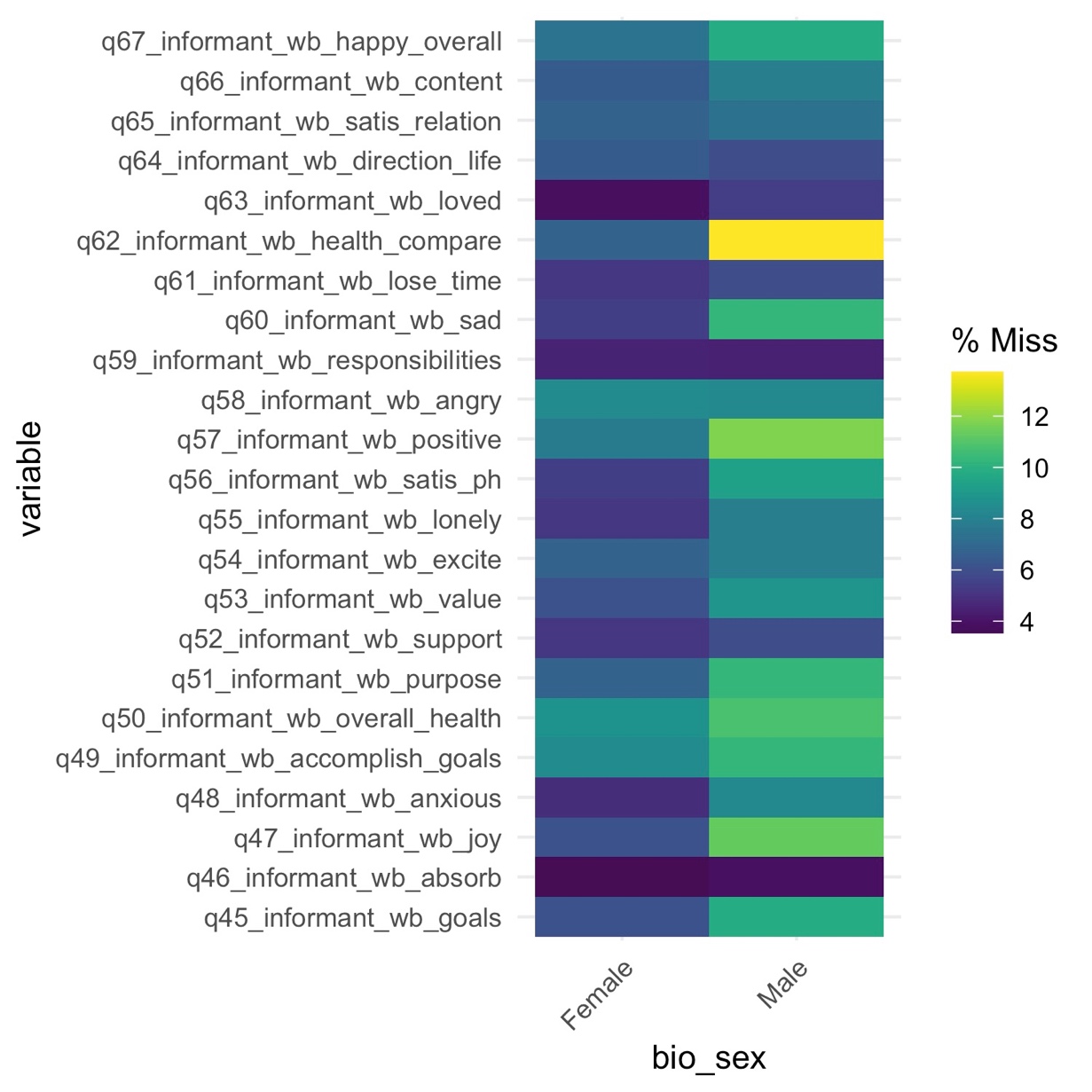

Supplement: Supplemental data [file Suppl_FigS1.docx]
