# Supplementary material for: Self-Report Measurement of Well-Being in Autistic Adults: Psychometric Properties of the PERMA Profiler
Source: Autism Adulthood. 2023 Dec 12;5(4):401–10. doi: 10.1089/aut.2022.0049 (PMC10726181; doi:10.1089/aut.2022.0049)
Supplement: Supplemental data [file Suppl_TableS2.docx]

**Table S2.** Five-factor model fit statistics for gender- and age-stratified CFA

| **Fit statistic/Index** | **Female/Woman**  **(N= 254)** | **Male/Man**  **(N= 188)** | **Age < 37**  **(N= 258)** | **Age >= 37**  **(N= 259)** |
| --- | --- | --- | --- | --- |
| RMSEA | 0.09 | 0.09 | 0.08 | 0.08 |
| TLI | 0.91 | 0.91 | 0.91 | 0.93 |
| CFI | 0.93 | 0.93 | 0.94 | 0.94 |
| SRMR | 0.05 | 0.06 | 0.05 | 0.06 |
