# Supplementary material for: Self-Report Measurement of Well-Being in Autistic Adults: Psychometric Properties of the PERMA Profiler
Source: Autism Adulthood. 2023 Dec 12;5(4):401–10. doi: 10.1089/aut.2022.0049 (PMC10726181; doi:10.1089/aut.2022.0049)
Supplement: Supplemental data [file Suppl_TableS3.docx]

**Table S3.** Correlation matrix for convergent and divergent validity of PERMA Profiler

|  | P | E | R | M | A | N | PH | O | AN | D | E |
| --- | --- | --- | --- | --- | --- | --- | --- | --- | --- | --- | --- |
| P |  |  |  |  |  |  |  |  |  |  |  |
| E | 0.54 |  |  |  |  |  |  |  |  |  |  |
| R | 0.65 | 0.39 |  |  |  |  |  |  |  |  |  |
| M | 0.74 | 0.43 | 0.62 |  |  |  |  |  |  |  |  |
| A | 0.57 | 0.34 | 0.47 | 0.68 |  |  |  |  |  |  |  |
| N | -0.44 | -0.14* | -0.29 | -0.36 | -0.23 |  |  |  |  |  |  |
| PH | 0.50 | 0.26 | 0.34 | 0.47 | 0.47 | -0.23 |  |  |  |  |  |
| O | 0.89 | 0.59 | 0.77 | 0.86 | 0.73 | -0.51 | 0.65 |  |  |  |  |
| AN | -0.17 | 0.00 | -0.10* | -0.13* | -0.16 | 0.33 | -0.20 | -0.20 |  |  |  |
| D | -0.38 | -0.24 | -0.24 | -0.33 | -0.28 | 0.37 | -0.25 | -0.42 | 0.38 |  |  |
| E | 0.67 | 0.36 | 0.58 | 0.62 | 0.5 | -0.35 | 0.48 | 0.72 | -0.17 | -0.3 |  |
| SAT | 0.72 | 0.45 | 0.57 | 0.69 | 0.53 | -0.33 | 0.45 | 0.76 | -0.11 | -0.31 | 0.82 |

P: Positive emotion; E: Engagement; R: Relationships; M: Meaning; A: Accomplishment; N: Negative emotion; PH: Physical health; O: Overall PERMA Well-being Score; AN: Anxiety; D: Depression; E: Thinks life is Excellent; SAT: Satisfied with life. All p<0.001 unless denoted by *(meaning p<0.05).
